# Vision-Related Quality of Life in Patients with Optic Neuropathy: Insights from a Portuguese Single Center Using the NEI-VFQ-25

**DOI:** 10.3390/neurolint17110184

**Published:** 2025-11-11

**Authors:** Sofia Bezerra, Ricardo Soares dos Reis, Maria José Sá, Joana Guimarães

**Affiliations:** 1 Department of Clinical Neurosciences and Mental Health, Faculty of Medicine, University of Porto, 4200-319 Porto, Portugal; 2 RISE-Health, Department of Clinical Neurosciences and Mental Health, Faculty of Medicine, University of Porto, 4200-319 Porto, Portugal; 3 Department of Neurology, São João University Hospital, 4200-319 Porto, Portugal; 4 School of Medicine and Biomedical Sciences, University Fernando Pessoa, 4249-004 Porto, Portugal; 5 RISE-Health, Faculty of Health Sciences, FP-BHS, FP-I3ID, University Fernando Pessoa, 4249-004 Porto, Portugal

**Keywords:** optic neuropathy, vision-related quality of life, NEI-VFQ-25, patient-reported outcomes

## Abstract

**Background/Objectives**: Optic neuropathies (ON) are a clinically heterogeneous group of disorders that can cause profound and lasting visual disability, with wide-ranging effects on patients’ quality of life. Although the NEI-VFQ-25 is an instrument for assessing vision-related quality of life (VRQoL), few studies have systematically compared patient-reported outcomes across multiple ON subtypes, especially in underrepresented populations. We aimed to delineate how etiological differences and longitudinal visual acuity trajectories shape VRQoL in a diverse Portuguese cohort with ON. **Methods**: This retrospective, cross-sectional study included 152 patients diagnosed with ON and followed at São João University Hospital, Portugal. All participants completed the validated NEI-VFQ-25. Diagnosis-specific differences in VRQoL were interrogated using ANCOVA and linear mixed-effects models, controlling for age and sex. Visual acuity changes over time were analyzed in relation to patient-reported outcomes. **Results**: Substantial heterogeneity in VRQoL was observed across ON subtypes. Patients with MS-related ON (MS-RON) and idiopathic ON reported significantly higher NEI-VFQ-25 scores in domains such as general vision, mental health, and dependency (F = 3.30, *p* = 0.013; ηp^2^ = 0.08), while those with ischemic or other inflammatory etiologies showed persistently lower scores. Notably, both final visual acuity and diagnosis were independently associated with NEI-VFQ-25 composite scores, highlighting the correlation between objective and subjective measures of visual function. Age and diagnosis independently predicted poorer VRQoL. **Conclusions**: This study provides the first comprehensive evaluation of vision-related quality of life (VRQoL) across a diverse cohort of optic neuropathy patients in a Portuguese tertiary center, using the NEI-VFQ-25. Our results underscore the heterogeneity of functional impact across ON subtypes, emphasizing the value of integrating sensitive, multidimensional assessment tools into neuro-ophthalmic clinical care, especially in populations historically underrepresented in research.

## 1. Introduction

Optic neuropathies (ON) represent a clinically heterogeneous group of disorders that result in varying degrees of visual impairment and disability, with etiologies ranging from glaucomatous, ischemic, isolated inflammatory episodes to systemic neuroimmunology diseases, such as multiple sclerosis (MS), neuromyelitis optica spectrum disorder (NMOSD), and myelin oligodendrocyte glycoprotein antibody-associated disease (MOGAD) [1,2,3]. While glaucoma is the most prevalent cause of optic neuropathy worldwide, particularly in general ophthalmology practice, demyelinating and ischemic forms are more commonly encountered in neuro-ophthalmology clinics and are the primary focus of this study. The functional deficits in ON extend beyond central visual acuity to encompass color discrimination, contrast sensitivity, and peripheral field loss, all of which profoundly affect vision-related quality of life (VRQoL) [4,5,6,7].

In recent years, the VRQoL has emerged as a critical, patient-centered outcome in neuro-ophthalmology. The National Eye Institute Visual Function Questionnaire-25 (NEI-VFQ-25) is widely used for this purpose in both ophthalmological and neuro-ophthalmological research [8,9]. Nonetheless, controversy remains regarding the best methods to quantify visual disability in ON, as NEI-VFQ-25 scores often diverge from traditional measures such as Snellen acuity or logMAR, underscoring the complex and subjective nature of visual dysfunction [10,11,12]. Furthermore, the specific contributions of non-acuity factors (e.g., contrast sensitivity, psychosocial burden) remain insufficiently characterized in diverse populations.

Despite an expanding international literature, most data derive from trial-based or registry cohorts with limited representation of certain ON subtypes and underrepresented populations [11,13,14,15,16,17,18]. However, comparisons of VRQoL across etiological subgroups, especially in Southern European and Portuguese-speaking cohorts, are scarce [19]. While a validated Portuguese version of the NEI-VFQ-25 exists, its psychometric performance in neuro-ophthalmological disease, and the patterns of VRQoL across ON subtypes, remain poorly defined.

Given these gaps, the present study aims to systematically evaluate VRQoL in a Portuguese tertiary referral hospital cohort with diverse ON etiologies, using the NEI-VFQ-25. We aimed to compare VRQoL scores across diagnostic groups and assess their association with demographic and longitudinal clinical variables, including changes in visual acuity over time during follow-up. By directly addressing the heterogeneity of patient experiences and integrating patient-reported outcomes, our findings provide new insights into the clinical evaluation and management of ON subtypes.

## 2. Materials and Methods

### 2.1. Study Design

This retrospective, cross-sectional study was conducted at São João University Hospital, a tertiary referral center in Porto, Portugal. Clinical records of all patients diagnosed with optic neuropathy (ON) between January 2004 and July 2023 were reviewed. Eligible patients were those still under active follow-up at the hospital, who were subsequently invited to participate during scheduled neurology or ophthalmology visits. The validated Portuguese version of the 25-Item National Eye Institute Visual Function Questionnaire (NEI-VFQ-25) was administered cross-sectionally via in-person interviews between September 2023 and September 2024. The study was approved by the institutional Ethics Committee (approval no. 331/2020), and all participants provided written informed consent in accordance with data protection regulations and the Declaration of Helsinki.

### 2.2. Participants and Diagnostic Criteria

Eligible participants were adults (≥18 years) at the time of ON diagnosis, confirmed according to standardized international criteria, and with availability for NEI-VFQ-25 administration during follow-up visits. To establish the diagnosis of ON, we adhered to the most up-to-date diagnostic criteria as follows: Multiple Sclerosis-related optic neuritis (MS-RON) was determined based on the 2017 revision of the McDonald Criteria [20]. Neuromyelitis optica spectrum disorder-related optic neuritis (NMOSD-RON) followed the “International consensus diagnostic criteria for Neuromyelitis Optica Spectrum Disorders”, with or without the presence of Aquaporin-4 Immunoglobulin (AQP4-IgG) status [21]. Myelin Oligodendrocyte Glycoprotein Antibody Disease (MOGAD-ON) was established in accordance with international criteria [2,3,22], requiring a clinical presentation compatible with ON and confirmed positivity for serum MOG-IgG using a cell-based assay, in the absence of alternative diagnoses. Chronic relapsing inflammatory ON (CRION) was diagnosed using the criteria proposed by Petzold and Plant through a systematic review [23], whereas Ischemic ON was defined according to Hayreh et al. [24]. The diagnosis of Idiopathic ON determined by excluding all other possible causes of optic neuritis [1,2,3], using the available methodology at the time of admission, and until the loss of follow-up. Our primary aim was to evaluate acquired, predominantly acute or subacute ON etiologies typically seen in neuro-ophthalmology practice, for which patient-reported outcome measures (PROMs) are most applicable. Exclusion criteria were defined to minimize heterogeneity and confounding, including: (1) Ocular comorbidities interfering with visual function assessment (e.g., dense cataract, corneal opacity, significant vitreous haze, or macular scarring); (2) High refractive error (myopia > 6 D; hyperopia or astigmatism > 3 D); (3) History or presence of glaucoma, or intraocular pressure > 30 mmHg; (4) Use of medications with known optic nerve toxicity (e.g., ethambutol, plaquenil, phenothiazines); (4) Traumatic optic neuropathy; (5) History of unrelated optic neuritis or optic disk pallor in the currently affected eye; (6) Hereditary optic neuropathy (e.g., Leber Hereditary Optic Neuropathy and Autosomal Dominant Optic Atrophy); owing to their distinct etiology, chronicity, and typical bilateral or abrupt onset, which contrast with acquired, usually unilateral and acute presentations; (7) History of unrelated optic neuritis or evidence of optic disk pallor in the currently affected eye, to ensure inclusion of only acute, incident ON cases; (8) Compressive optic neuropathies (e.g., meningioma, pituitary adenoma), as these conditions typically have chronic, progressive courses and are managed surgically or oncologically, introducing additional confounding factors for quality-of-life outcomes. These criteria were implemented to minimize diagnostic heterogeneity and eliminate confounding factors, ensuring the comparability and internal validity of patient-reported outcomes across groups.

We stratified the follow-up current diagnosis in five ON categories [1,2,3,20,21,22,23,24]: Multiple sclerosis-related optic neuritis (MS-RON); Other demyelinating/inflammatory ON (DON/ION)—includes Neuromyelitis Optica Spectrum Disorder (NMOSD), MOG Antibody Disease (MOGAD) and Chronic Relapsing Inflammatory Optic Neuritis (CRION); Idiopathic ON; Ischemic ON, encompassing both Arteritic Anterior Ischemic Optic Neuropathy (AAION)—including cases secondary to giant cell arteritis (GCA) and other systemic vasculitis (e.g., lupus, polyarteritis nodosa), and Non-Arteritic Anterior Ischemic Optic Neuropathy (NAION); and Other ON, that includes toxic, nutritional and infectious. The rationale for these groupings follows recent international guidelines [1,2,3,20,21,22,23,24], but we acknowledge the heterogeneity, particularly within the Other ON group, as a limitation. Toxic optic neuropathies included in the Other ON group were limited to cases attributable to chronic alcohol abuse or severe nutritional deficiency, where no medication or acute toxin-induced ON was documented. Smoking-related ON was not present in our cohort. Cases of ON related to active or historical use of medications with known toxicity to optic nerves were excluded. Due to the small sample sizes of individual subgroups within the Ischemic ON and Other ON categories, further statistical comparisons between subtypes were not performed. However, we provide a descriptive sample size (n) of these subgroups in Supplementary Table S1.

Out of 173 screened patients, 21 were excluded based on the criteria listed above, resulting in a final cohort of 152 participants. These exclusions ensured diagnostic homogeneity and minimized bias from confounding structural, pharmacological, or traumatic factors.

### 2.3. Clinical Variables and NEI-VFQ-25 Administration

Best-corrected visual acuity (BCVA) of the affected eye was extracted from medical records at the following timepoints: admission, discharge, one year post-onset, and last available follow-up. Snellen BCVA values were converted to logarithm of the minimum angle of resolution (logMAR), including conversion of qualitative measures (“counting fingers”, “hand motion”, “light perception”, “no light perception”) [25,26]. All patients completed the Portuguese NEI-VFQ-25, which comprises 25 items distributed across 12 domains (one for general health, 11 vision-targeted subscales: general vision, ocular pain, near and distance vision, social functioning, mental health, role difficulties, dependency, driving, color vision, and peripheral vision). The scoring and conversion to a 0–100 scale were performed according to the validated scoring manual (RAND Manual). The original questionnaire and scoring manual are available in Supplementary Materials. Clinical variables such as BCVA, color vision, and peripheral vision were extracted from medical records and are presented in their native formats. Responses were transformed to a 0–100 scale, with higher scores indicating better vision-related function, and the composite score was calculated as the mean of vision-targeted subscales (excluding general health). The time from ON diagnosis to NEI-VFQ-25 questionnaire administration was recorded for all patients, and detailed statistics are provided in Section 3. Although a 10-item neuro-ophthalmology supplement to the NEI-VFQ-25 exists [27], it was not administered due to the absence of a validated Portuguese version. The inclusion of an unvalidated translation would risk compromising the instrument’s psychometric integrity [28,29]. This limitation highlights the need for future validation studies tailored to Portuguese-speaking neuro-ophthalmology populations.

### 2.4. Statistical Analyses

Analyses were performed using R software (version 4.2.3) [30]. Descriptive statistics are reported as means (standard deviation) for continuous variables and proportions for categorical variables. ANCOVA was used to compare NEI-VFQ-25 subscale and composite scores across ON diagnostic categories, adjusting for age and sex, with Bonferroni correction for multiple testing. Effect sizes were reported as partial eta squared (ηp^2^). Longitudinal changes in logMAR were modeled using linear mixed-effects models (LMMs) [31], including fixed effects for timepoint, age, sex, diagnosis, and their interactions, with a random intercept for patient ID to account for repeated measures. Model assumptions (linearity, normality of residuals, homoscedasticity, multicollinearity) were systematically checked and met. Statistical significance was set at *p* < 0.05.

## 3. Results

The study included 152 patients aged 19 to 90 years, with a mean age of 48.24 (SD = 16.96), mostly males (n = 100, 65.8%). The distribution of the affected eyes showed that 47.4% had right-eye involvement (n = 72), 38.2% had left-eye involvement (n = 55), and 14.5% had bilateral involvement (n = 22). The median duration of the questionnaire application was 26.0 months (Q1 = 8.0, Q3 = 92), and the median duration of follow-up was 29.5 months (Q1 14.0, Q3 = 62.0).

The diagnostic profiles of the patients were diverse. MS-RON was the most prevalent diagnosis, observed in 61 patients (40.1%), followed by Ischemic ON (n = 34; 22.3%), Idiopathic ON (n = 30; 19.7%), other demyelinating or inflammatory ON (n = 20; 13.2%), and other ON (n = 7; 4.6%).

### 3.1. NEI-VFQ-25 Scores by Diagnosis

Table 1 shows the results of ANCOVAs for NEI VFQ-25 score comparisons by diagnostically adjusted age and sex. Regarding general health, the results indicated that there were no statistically significant differences among the different ON subtypes (F = 0.65, *p* = 0.631, ηp^2^ = 0.02).

The general vision score was significantly higher in patients with Idiopathic ON or MS than in those with other diagnoses (F = 3.30, *p* = 0.013, ηp^2^ = 0.08). The mental health score was significantly higher in patients with MS-RON than in those with Ischemic and Other ON diagnoses (F = 4.53, *p* = 0.002, ηp^2^ = 0.11). The dependency score was significantly higher in patients with MS than in those with other diagnoses (F = 2.74, *p* = 0.031, ηp^2^ = 0.07). The color vision score was significantly lower for other diagnostics than for idiopathic ON, MS, and ischemic ON diagnostics (F = 6.23, *p* < 0.001, ηp^2^ = 0.15). Peripheral vision score was significantly higher in patients with MS than in those with other diagnoses (F = 3.47, *p* = 0.010, ηp^2^ = 0.09). The total score was significantly higher for patients with MS than for those with other diagnoses (F = 3.73, *p* = 0.006, ηp^2^ = 0.09). Age was significantly associated with lower scores on all measures. Males scored significantly higher on general vision than females (F = 4.41, *p* = 0.038, ηp^2^ = 0.03).

### 3.2. Longitudinal Visual Acuity

**Figure 1:**
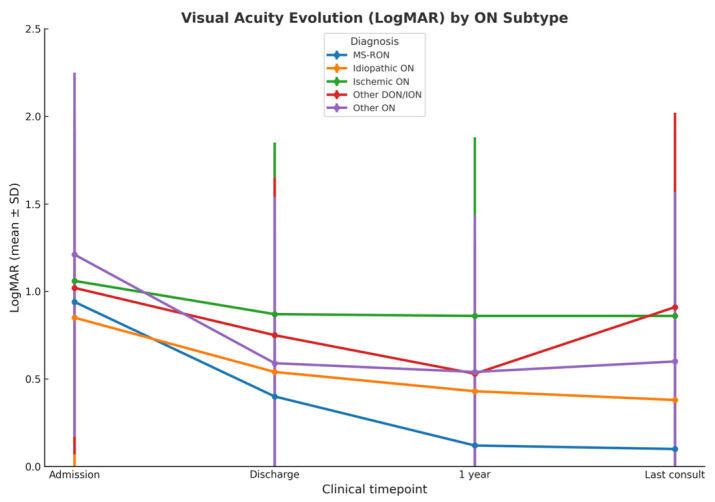
Adjusted means and 95% CI of VA (LogMAR) across time by diagnostic.

Figure 1 displays the adjusted mean LogMAR values with standard deviations (±SD) over four clinical timepoints (admission, discharge, 1 year, and last consult) for different ON subtypes. A decrease in LogMAR reflects visual improvement. Among the subgroups, MS-related ON (MS-RON) demonstrated the most pronounced and consistent recovery in visual acuity, showing significantly greater improvement compared to all timepoints of Ischemic ON and to the final follow-up of other demyelinating/inflammatory conditions, as detailed in Table 2, and represented in Figure 1.

Table 3 presents the results of a linear mixed model (LMM) with visual acuity (LogMAR) as the outcome variable, incorporating fixed effects for time, eye, sex, age, and the interaction of time with diagnosis, along with a random intercept term for the ID variable to account for the correlation between repeated measurements of the LogMAR outcome within the same individual (ID). The model was adjusted in accordance with the recommendations of Bates et al. [20]. Time negatively impacted comparisons between admission and discharge (β = −0.53, *p* < 0.001), one year after discharge (β = −0.83, *p* < 0.001), and the last consultation (β = −0.86, *p* < 0.001). Age positively influenced LogMAR (β = 0.01, *p* = 0.010). Compared with MS, the interaction between time and diagnosis was statistically significant for one year × ischemic (β = 0.65, *p* < 0.001), last consultation × ischemic (β = 0.67, *p* < 0.001), and last consultation × NMOSD (β = 0.71, *p* < 0.001).

Although visual acuity means and standard deviations between diagnostic groups showed some overlap, the longitudinal analysis using linear mixed-effects models revealed statistically significant differences in recovery trajectories. Specifically, compared with the MS-RON group, both the ischemic ON and other demyelinating/inflammatory ON subgroups exhibited less improvement in visual acuity at one year and at last follow-up (1 year × ischemic ON: β = 0.65, *p* < 0.001; last consult × ischemic ON: β = 0.67, *p* < 0.001; last consult × other DON/ION: β = 0.71, *p* < 0.001), as shown above in Table 3. These results indicate that, despite some overlap in standard deviations, the recovery pattern observed in the MS-RON group is statistically robust and not merely a descriptive trend.

### 3.3. Association Between Final Visual Acuity and VRQoL

A linear mixed-effects model (LMM) was performed to examine the association between final best-corrected visual acuity (logMAR at last follow-up; lower values indicate better vision) and the NEI-VFQ-25 composite score, adjusted for age and diagnostic group (MS group as reference). Sex was not a significant predictor and was omitted for clarity. The coefficients and full results of the LMM are presented in Table 4.

The analysis demonstrated a statistically significant negative association between logMAR and the composite score [(β = −13.2, 95% CI: [−18.7 to −7.7], *p* < 0.001)], demonstrating that poorer visual acuity (Higher logMAR values indicate worse vision) is significantly associated with lower VRQoL scores (*p* < 0.001). This relationship remained significant after adjusting for diagnosis category, with MS-ON patients exhibiting both the best visual outcomes and the highest VRQoL scores among all groups and less pronounced among ischemic and NMOSD/MOGAD subgroups.

## 4. Discussion

Optic neuropathies (ON) represent a broad spectrum of etiologies with variable clinical courses and functional outcomes, often resulting in profound impact on vision and quality of life. The growing recognition that the lived experience of visual impairment extends far beyond Snellen acuity or structural measures has propelled vision-related quality of life (VRQoL) to the forefront of neuro-ophthalmological research [4,11,12]. The NEI-VFQ-25 questionnaire has become the standard for assessing patient-reported visual function, yet its application in ON populations, particularly in Portuguese-speaking contexts, remains limited [11].

The findings revealed several noteworthy results that have important implications for clinical management and understanding of vision-related quality of life in patients with different ON diagnoses. In this study, we comprehensively assessed VRQoL among patients with diverse ON etiologies, confirming and extending several findings from previous cohorts. Our data demonstrate that MS-related optic neuritis (MS-RON) is associated with more favorable VRQoL scores across multiple domains when compared to other demyelinating, ischemic, or idiopathic ON, consistent with reports from international studies [11,15,16,17,32] and the Optic Neuritis Treatment Trial (ONTT) [33]. In particular, MS-RON patients reported superior function in general vision, near activities, and mental health, reflecting not only better visual recovery, but also greater adaptation and autonomy in daily life over time. Similarly, the idiopathic ON group, though less extensively characterized in the literature, showed an intermediate profile, frequently paralleling MS-RON in subjective scores, which may reflect a milder phenotype or underrecognized MOG-antibody disease [1,3,11].

In contrast, patients with NMOSD, MOGAD, and CRION consistently reported lower VRQoL, especially in mental health, role difficulty, and dependency domains, highlighting substantial psychosocial and functional burdens. These results are supported by recent multicenter analyses which underscore age-related decline and the unpredictable, relapsing nature of these conditions [17,18,34,35]. Ischemic ON, meanwhile, was associated with persistently low scores in near vision and social functioning, consistent with the ischemic damage, irreversible axonal loss and poor recovery reported in prior studies [36,37,38,39].

An important observation in our cohort is that, while MS-ON patients exhibited both superior final visual acuity and higher VRQoL composite scores compared to other diagnostic groups, our regression analysis (LMM, adjusted for age) revealed a statistically significant association between final logMAR and NEI-VFQ-25 composite scores for all groups. In addition, all non-MS diagnostic categories showed significantly lower VRQoL composite scores compared to the MS group (all *p* < 0.05).

These finding highlights that, although patient-reported outcomes such as VRQoL capture a broader and more nuanced spectrum of disability, including psychosocial adaptation, mental health, and functional independence; objective measures of visual function remain closely linked to subjective perceptions of quality of life, particularly in those with more favorable visual recovery, such as MS patients. These results underscore the importance of integrating both objective and subjective outcome measures in the clinical evaluation and management of optic neuropathies.

Importantly, our results also reinforce previous evidence [8,9,27,40,41] that diagnosis-specific factors, psychosocial domains, and functional adaptation significantly contribute to overall VRQoL, especially in those with persistent symptoms despite relatively good visual acuity. Thus, although the association between visual acuity and quality of life is significant, it does not fully account for the heterogeneity in patient-reported outcomes across ON subtypes, supporting the use of multidimensional assessment tools such as the NEI-VFQ-25 to guide rehabilitation and patient-centered care.

Our results are consistent with recent studies that have compared QoL in various ON subtypes. Notably, the work of Nicolescu et al. [35] in NMOSD and MOGAD, and Gassan et al. [17], reinforce the impact of disease etiology and aging on VRQoL. Song et al. [18] demonstrated the high burden of depression and functional impairment in AQP4-IgG seropositive ON, which aligns with our findings of diminished mental health in other inflammatory ON groups. In non-arteritic ischemic ON, Su et al. [36] found significant VRQoL compromise, paralleling our results in this group. Furthermore, the integration of our findings with other studies on visual function and quality of life in different ocular and systemic contexts—such as the impact of ocular syphilis [14], atypical ON [16], and broader reviews of vision-targeted PROMs [4,11,12], underscores the universal importance of a multidimensional, patient-centered approach in both clinical care and research.

This study has certain limitations inherent to its retrospective, single-center design, as well as the long inclusion period from 2004 to 2023, during which time, significant advancements occurred in neuro-ophthalmological diagnostics and data management. The incorporation of structural correlates, particularly optical coherence tomography (OCT) derived retinal nerve fiber layer (RNFL) thickness, was inherently limited by the retrospective design and the broad temporal period of this study. Much of the cohort pre-dated the widespread adoption of electronic health records and routine OCT imaging, resulting in substantial variability and frequent absence of historical structural data, especially among patients seen in earlier years. Although previous studies have highlighted robust correlations between RNFL thinning and VRQoL in MS and NMOSD [17,41,42], our findings reaffirm that patient-reported outcome measures (PROMs) capture unique and clinically meaningful aspects of disability that are complementary, but not redundant, to objective imaging [40,41]. Similarly, the lack of consistent visual field and low-contrast sensitivity assessments reflects both the constraints of retrospective clinical research and the evolution of neuro-ophthalmic practice. The established prognostic and functional value of low-contrast acuity in ON and MS should be integrated into future prospective studies [43,44]. Additionally, the lack of a validated Portuguese version of the NEI-VFQ-25 neuro-ophthalmic supplement prevented its inclusion, reflecting broader challenges in cross-cultural instrument validation, but also highlights the need for future prospective and multicenter research.

Notwithstanding these limitations, this study represents the most comprehensive assessment to date of vision-related quality of life across a diverse cohort of optic neuropathy patients in a Portuguese tertiary center. By systematically comparing subdomain scores among distinct etiological subgroups, we demonstrated that patient-reported outcomes capture clinically meaningful differences often missed by traditional visual acuity metrics. Our results underscore the heterogeneity of functional impact across ON subtypes, emphasizing the value of integrating sensitive, multidimensional assessment tools into routine clinical care. Importantly, these findings advocate for a broader, patient-centered approach to management and highlight subgroups at risk for disproportionate functional burden. This work offers a framework for future prospective and multicenter studies, aiming to refine outcome measurement and advance personalized care in neuro-ophthalmology.

## Figures and Tables

**Table 1:** ANCOVAs for Scores NEI VFQ-25 comparisons by diagnostic adjusted for age and gender.

						F-Test		
NEI-VFQ-25 Variables	MS-RON (n = 61)	Idiopathic ON (n = 30)	Ischemic ON (n = 34)	Other DON ^a^ ION ^b^ (n = 20)	Other ON (n = 7)	Diagnostic	Age	Gender
General Health	40.87 (3.41)	36.68 (4.19)	46.33 (5.08)	43.98 (5.18)	38.12 (7.98)	F = 0.65, *p* = 0.631, ηp^2^ = 0.02	F = 33.32, *p* < 0.001, ηp^2^ = 0.19 *	F = 3.39, *p* = 0.068, ηp^2^ = 0.02
General Vision	69.75 (2.42)	68.16 (2.98)	58.67 (3.61)	63.82 (3.68)	49.68 (5.68)	F = 3.30, *p* = 0.013, ηp^2^ = 0.08 * (a)	F = 21.62, *p* < 0.001, ηp^2^ = 0.13 *	F = 4.41, *p* = 0.038, ηp^2^ = 0.03 *
Ocular Pain	69.49 (3.41)	59.55 (4.20)	75.03 (5.09)	68.51 (5.19)	66.79 (8.00)	F = 1.67, *p* = 0.161, ηp^2^ = 0.04	F = 10.23, *p* = 0.002, ηp^2^ = 0.07 *	F = 0.03, *p* = 0.866, ηp^2^ = 0.00
Near Activities	79.32 (3.30)	65.71 (4.07)	71.48 (4.92)	62.30 (5.02)	65.55 (7.74)	F = 2.99, *p* = 0.021, ηp^2^ = 0.08 * (e)	F = 36.30, *p* < 0.001, ηp^2^ = 0.20 *	F = 0.16, *p* = 0.693, ηp^2^ = 0.00
Distance Activities	83.66 (3.30)	78.89 (4.05)	77.19 (4.91)	72.72 (5.01)	59.95 (7.72)	F = 2.36, *p* = 0.056, ηp^2^ = 0.06	F = 18.60, *p* < 0.001, ηp^2^ = 0.11 *	F = 1.52, *p* = 0.220, ηp^2^ = 0.01
Social Functioning	95.78 (3.05)	90.58 (3.77)	85.23 (4.55)	83.20 (4.64)	77.64 (7.15)	F = 2.16, *p* = 0.076, ηp^2^ = 0.06	F = 6.73, *p* = 0.010, ηp^2^ = 0.04 *	F = 0.00, *p* = 0.950, ηp^2^ = 0.00
Mental Health	71.29 (3.15)	61.58 (3.87)	52.30 (4.69)	56.53 (4.78)	41.86 (7.37)	F = 4.53, *p* = 0.002, ηp^2^ = 0.11 * (b)	F = 18.87, *p* < 0.001, ηp^2^ = 0.12 *	F = 0.50, *p* = 0.480, ηp^2^ = 0.00
Role Difficulties	82.77 (4.47)	71.54 (5.50)	60.63 (6.66)	65.85 (6.80)	53.96 (10.48)	F = 2.54, *p* = 0.042, ηp^2^ = 0.07 * (e)	F = 13.82, *p* < 0.001, ηp^2^ = 0.09 *	F = 0.01, *p* = 0.913, ηp^2^ = 0.00
Dependency	89.16 (3.87)	80.28 (4.76)	76.08 (5.76)	73.19 (5.87)	60.67 (9.6)	F = 2.74, *p* = 0.031, ηp^2^ = 0.07 * (c)	F = 18.44, *p* < 0.001, ηp^2^ = 0.11 *	F = 0.05, *p* = 0.832, ηp^2^ = 0.00
Driving	78.88 (4.31)	63.60 (5.45)	69.93 (9.02)	68.28 (7.21)	53.06 (9.74)	F = 2.03, *p* = 0.096, ηp^2^ = 0.07	F = 14.10, *p* < 0.001, ηp^2^ = 0.12 *	F = 3.39, *p* = 0.068, ηp^2^ = 0.03
Color vision	94.85 (2.94)	92.46 (3.63)	98.31 (4.43)	81.35 (4.48)	65.67 (6.92)	F = 6.23, *p* < 0.001, ηp^2^ = 0.15 * (d)	F = 9.00, *p* = 0.003, ηp^2^ = 0.06 *	F = 2.14, *p* = 0.145, ηp^2^ = 0.01
Peripheral Vision	80.78 (3.95)	70.28 (4.82)	60.82 (5.85)	65.30 (6.11)	47.35 (9.17)	F = 3.47, *p* = 0.010, ηp^2^ = 0.09 * (c)	F = 9.58, *p* = 0.002, ηp^2^ = 0.06 *	F = 0.55, *p* = 0.461, ηp^2^ = 0.00
NEI-VFQ-25 Composite	81.39 (2.72)	72.91 (3.34)	70.97 (4.05)	68.30 (4.13)	58.27 (6.36)	F = 3.73, *p* = 0.006, ηp^2^ = 0.09 * (c)	F = 24.10, *p* < 0.001, ηp^2^ = 0.14 *	F = 0.33, *p* = 0.569, ηp^2^ = 0.02

* Statistically significant at *p* < 0.05; results presented as adjusted means and standard errors; Bonferroni multiple comparisons tests found significant differences at *p* < 0.05: (a) other ON vs. Idiopathic ON and MS-RON; (b) MS-RON vs. ischemic ON and other ON; (c) MS-RON vs. other ON; (d) other ON vs. Idiopathic ON, MS-RON and Ischemic ON (e) no differences after Bonferroni correction; ηp^2^, partial eta squared. Abbreviation: ^a^ DON = Demyelinating Optic neuropathy; ^b^ ION = Inflammatory Optic Neuropathy.

**Table 2:** Longitudinal changes in Mean (SD) LogMAR visual acuity across ON Subtypes at four clinical timepoints.

Diagnostic	Admission	Discharge	1 Year	Last Consult
MS-RON	0.94 (0.97)	0.40 (1.40)	0.12 (0.33)	0.10 (0.31)
Idiopathic ON	0.85 (0.94)	0.54 (0.80)	0.43 (0.87)	0.38 (0.76)
Ischemic ON	1.06 (0.99)	0.87 (0.98)	0.86 (1.02)	0.86 (1.05)
Other DON/ION	1.02 (0.95)	0.75 (0.90)	0.53 (0.75)	0.91 (1.11)
Other ON	1.21 (1.04)	0.59 (0.95)	0.54 (0.9)	0.60 (0.97)

**Table 3:** LMM for LogMAR across time adjusted for time, eye, sex, age, and interaction of time with diagnostic.

Variable	Estimate	Std. Error	t Value	95% CI	*p*-Value
(Intercept)	0.65	0.21	3.12	(0.25; 1.05)	<0.001 ***
Time (reference = admission)					
Discharge	−0.53	0.13	−3.98	(−0.78; −0.27)	<0.001 ***
1 year	−0.83	0.13	−6.30	(−1.09; −0.58)	<0.001 ***
Last appointment	−0.86	0.13	−6.53	(−1.12; −0.61)	<0.001 ***
Diagnostic (reference = MS)					
Idiopathic ON	−0.24	0.21	−1.18	(−0.64; 0.15)	0.240
Ischemic ON	−0.25	0.24	−1.04	(−0.72; 0.21)	0.300
Other DON/ION	−0.17	0.25	−0.68	(−0.65; 0.31)	0.500
Other ON	0.20	0.32	0.63	(−0.41; 0.81)	0.530
Eye (reference = Left)					
Right	−0.13	0.08	−1.61	(−0.29; 0.03)	0.110
Gender (reference = female)					
Male	−0.06	0.12	−0.51	(−0.30; 0.17)	0.610
Age	0.01	0.00	2.47	(0.00; 0.02)	0.010 **
Time × Diagnostic (reference= time × MS)					
Discharge × Idiopathic ON	0.21	0.21	1.00	(−0.20; 0.62)	0.320
1 year × Idiopathic ON	0.39	0.22	1.84	(−0.02; 0.81)	0.070
Last consult × Idiopathic ON	0.38	0.21	1.78	(−0.03; 0.79)	0.080
Discharge × Ischemic ON	0.34	0.21	1.62	(−0.07; 0.75)	0.110
1 year × Ischemic ON	0.65	0.21	3.06	(0.24; 1.06)	<0.001 ***
Last consult × Ischemic	0.67	0.21	3.15	(0.26; 1.08)	<0.001 ***
Discharge × Other DON/ION	0.26	0.25	1.01	(−0.23; 0.74)	0.310
1 year × Other DON/ION	0.37	0.25	1.46	(−0.12; 0.85)	0.140
Last consult × Other DON/ION	0.71	0.25	2.88	(0.24; 1.19)	<0.001 ***
Discharge × Other	−0.09	0.31	−0.30	(−0.70; 0.51)	0.760
1 year × Other	0.16	0.31	0.52	(−0.44; 0.77)	0.610
Last consult × Other	0.26	0.33	0.78	(−0.38; 0.89)	0.440

** *p* < 0.01; *** *p* < 0.001.

**Table 4:** LMM for the association between logMAR and NEI-VFQ-25 composite scores.

Variable	Estimate (β)	Std. Error	t-Value	95% CI	*p*-Value
(Intercept, MS group)	90.1	3.0	30.1	84.2–96.0	<0.001 *
logMAR (final, continuous)	−13.2	2.8	−4.71	−18.7–−7.7	<0.001 *
Age (per year)	−0.23	0.07	−3.29	−0.37–−0.09	0.001 *
Idiopathic ON	−7.6	3.4	−2.24	−14.3–−0.9	0.027 *
Ischemic ON	−21.1	4.1	−5.15	−29.2–−13.0	<0.001 *
Other Demyelinating/ION	−19.3	5.0	−3.86	−29.2–−9.4	<0.001 *
Other ON	−19.8	8.1	−2.45	−36.0–−3.6	0.016 *

* Statistically significant at *p* < 0.05.

## Data Availability

All data presented in this paper are available in the manuscript or from the corresponding author.

## Supplementary Materials

The following supporting information can be downloaded at: https://www.mdpi.com/article/10.3390/neurolint17110184/s1, Table S1: Number of cases (n) for each etiological subgroup within the Ischemic and Other ON categories; Questionnaire: National Eye Institute Visual Functioning Questionnaire-25 (VFQ-25)—version 2000; NEI VFQ-25 Scoring Algorithm—August 2000.

## Abbreviations

The following abbreviations are used in this manuscript:

NEI-VFQ-25	The National Eye Institute Visual Function Questionnaire-25
VRQoL	Vision- Related Quality of Life
ON	Optic Neuropathy
MS-RON	Multiple Sclerosis- Related Optic Neuritis
DON/ION	Demyelinating/Inflammatory Optic Neuropathy
MOGAD	MOG Antibody Disease
CRION	Chronic Relapsing Inflammatory Optic Neuritis
NMSOD	Neuromyelitis Optica spectrum disorder

## References

[1] Galetta S.L. (2022). A new classification for diagnosis of optic neuritis. Lancet Neurol..

[2] Ducloyer J.B., Marignier R., Wiertlewski S., Lebranchu P. (2021). Optic neuritis classification in 2021. Eur. J. Ophthalmol..

[3] Petzold A., Fraser C.L., Abegg M., Alroughani R., Alshowaeir D., Alvarenga R., Andris C., Asgari N., Barnett Y., Battistella R. (2022). Diagnosis and classification of optic neuritis. Lancet Neurol..

[4] Panthagani J., O’Donovan C., Aiyegbusi O.L., Liu X., Bayliss S., Calvert M., Pesudovs K., Denniston A.K., Moore D.J., Braithwaite T. (2023). Evaluating patient-reported outcome measures (PROMs) for future clinical trials in adult patients with optic neuritis. Eye.

[5] Schmidt F., Zimmermann H., Mikolajczak J., Oertel F.C., Pache F., Weinhold M., Schinzel J., Bellmann-Strobl J., Ruprecht K., Paul F. (2017). Severe structural and functional visual system damage leads to profound loss of vision-related quality of life in patients with neuromyelitis optica spectrum disorders. Mult. Scler. Relat. Disord..

[6] Demmin D., Silverstein S.M. (2020). Visual Impairment and Mental Health: Unmet Needs and Treatment Options. Clin. Ophthalmol..

[7] Okrent Smolar A.L., Gagrani M., Ghate D. (2022). Peripheral visual field loss and activities of daily living. Curr. Opin. Neurol..

[8] Mangione C.M., Lee P.P., Pitts J., Gutierrez P., Berry S., Hays R.D. (1998). Psychometric Properties of the National Eye Institute Visual Function Questionnaire (NEI-VFQ). Arch. Ophthalmol..

[9] Mangione C.M., Lee P.P., Gutierrez P.R., Spritzer K., Berry S., Hays R.D. (2001). Development of the 25-Item National Eye Institute Visual Function Questionnaire. JAMA Ophthalmol..

[10] Goldstein J.E., Bradley C., Gross A.L., Jackson M., Bressler N.M., Massof R.W. (2022). The NEI VFQ-25C: Calibrating Items in the National Eye Institute Visual Function Questionnaire-25 to Enable Comparison of Outcome Measures. Transl. Vis. Sci. Technol..

[11] Assi L., Chamseddine F., Ibrahim P., Sabbagh H., Rosman L., Congdon N., Evans J., Ramke J., Kuper H., Burton M.J. (2021). A Global Assessment of Eye Health and Quality of Life: A Systematic Review of Systematic Reviews. JAMA Ophthalmol..

[12] Vélez C.M., Bernal Ramírez P., Oviedo-Cáceres Mdel P., Lugo Agudelo L.H., Posada A.M., Hernández-Padilla M.L., Astudillo Valverde E., Suárez-Escudero J.C. (2022). Psychometric Properties of Scales for Assessing the Vision-related Quality of Life of People with Low Vision: A Systematic Review. Ophthalmic Epidemiol..

[13] Ramin S., Rostami F., Ahmadieh H., Daftarian N., Nourinia R., Abbasi A., Kheiri B., Sabbaghi H., Sheibani K. (2024). Vision-related quality of life in patients with retinal vein occlusion. Int. Ophthalmol..

[14] Silva M.S.F., Arantes T.E., Moreto R., Smith J.R., Furtado J.M. (2023). Vision-related quality of life in patients treated for ocular syphilis. Sci. Rep..

[15] Balcer L.J., Miller D.H., Reingold S.C., Cohen J.A. (2015). Vision and vision-related outcome measures in multiple sclerosis. Brain.

[16] Jiang Z., Qian H., Wei S. (2022). Vision-related quality of life in patients with atypical optic neuritis. Front. Pain Res..

[17] Gassan A.A., Konig A., Nisenbaum R., Freedman M.S., Lee L., Marrie R.A., McCombe J.A., Micieli J., Morrow S.A., Parks N.E. (2025). Comparison of vision-related quality of life in NMOSD and MOGAD. Mult. Scler. Relat. Disord..

[18] Song R., Huang W., Yang J., Tang X., Huang Y., Chen Y., Zhao M., Hu Q., Du Y. (2023). Association of aquaporin-4 antibody-seropositive optic neuritis with vision-related quality of life and depression. Front. Neurol..

[19] Abe R.Y., Medeiros F.A., Davi M.A., Gonçalves C., Bittencourt M.D., Roque A.B., Boccato J., Costa V.P., de Vasconcellos J.P.C. (2019). Psychometric properties of the Portuguese version of the National Eye Institute Visual Function Questionnaire-25. PLoS ONE.

[20] Thompson A.J., Banwell B.L., Barkhof F., Carroll W.M., Coetzee T., Comi G., Correale J., Fazekas F., Filippi M., Freedman M.S. (2018). Diagnosis of multiple sclerosis: 2017 revisions of the McDonald criteria. Lancet Neurol..

[21] Wingerchuk D.M., Banwell B., Bennett J.L., Cabre P., Carroll W., Chitnis T., de Seze J., Fujihara K., Greenberg B., Jacob A. (2015). International consensus diagnostic criteria for neuromyelitis optica spectrum disorders. Neurology.

[22] Bennett J.L., Costello F., Chen J.J., Petzold A., Biousse V., Newman N.J., Galetta S.L. (2022). Optic neuritis and autoimmune optic neuropathies: Advances in diagnosis and treatment. Lancet Neurol..

[23] Petzold A., Plant G.T. (2014). Chronic relapsing inflammatory optic neuropathy: A systematic review of 122 cases reported. J. Neurol..

[24] Hayreh S.S. (2011). Management of ischemic optic neuropathies. Indian J. Ophthalmol..

[25] Schulze-Bonsel K., Feltgen N., Burau H., Hansen L., Bach M. (2006). Visual acuities “hand motion” and “counting fingers” can be quantified with the Freiburg Visual Acuity Test. Investig. Ophthalmol. Vis. Sci..

[26] Moussa G., Bassilious K., Mathews N. (2021). A novel excel sheet conversion tool from Snellen fraction to LogMAR including “counting fingers”, “hand movement”, “light perception” and “no light perception” and focused review of literature of low visual acuity reference values. Acta Ophthalmol..

[27] Raphael B.A., Galetta K.M., Jacobs D.A., Markowitz C.E., Liu G.T., Nano-Schiavi M.L., Galetta S.L., Maguire M.G., Mangione C.M., Globe D.R. (2006). Validation and test characteristics of a 10-item neuro-ophthalmic supplement to the NEI-VFQ-25. Am. J. Ophthalmol..

[28] Wild D., Grove A., Martin M., Eremenco S., McElroy S., Verjee-Lorenz A., Erikson P. (2005). Principles of Good Practice for the Translation and Cultural Adaptation Process for Patient-Reported Outcomes (PRO) Measures: Report of the ISPOR Task Force for Translation and Cultural Adaptation. Value Health.

[29] Pesudovs K., Burr J.M., Harley C., Elliott D.B. (2007). The development, assessment, and selection of questionnaires. Optom. Vis. Sci..

[30] R Core Team (2023). R: A Language and Environment for Statistical Computing.

[31] Bates D., Mächler M., Bolker B., Walker S. (2015). Fitting Linear Mixed-Effects Models Using lme4. J. Stat. Softw..

[32] Villoslada P., Solana E., Alba-Arbalat S., Martinez-Heras E., Vivo F., Lopez-Soley E., Calvi A., Camos-Carreras A., Dotti-Boada M., Bailac R.A. (2024). Retinal Damage and Visual Network Reconfiguration Defines Visual Function Recovery in Optic Neuritis. Neurol. Neuroimmunol. Neuroinflamm..

[33] Cleary P.A., Beck R.W., Bourque L.B., Backlund J.C., Miskala P.H. (1997). Visual symptoms after optic neuritis. Results from the Optic Neuritis Treatment Trial. J. Neuro-Ophthalmol..

[34] Sotirchos E.S., Filippatou A., Fitzgerald K.C., Salama S., Pardo S., Wang J., Ogbuokiri E., Cowley N.J., Pellegrini N., Murphy O.C. (2020). Aquaporin-4 IgG seropositivity is associated with worse visual outcomes after optic neuritis than MOG-IgG seropositivity and multiple sclerosis, independent of macular ganglion cell layer thinning. Mult. Scler. J..

[35] Nicolescu M., Häußler V., Paul F., Oertel F.C., Schindler P., Strobl J.B., Krumbholz M., Hümmert M.W., Bütow F., Tkachenko D. (2024). Visual quality of life in NMOSD and MOGAD: Profiles, dynamics and associations with ageing and vision. J. Neurol..

[36] Su Y., Bai G., Tian H., Zhang S., Liu Y., Zhang G., Liu L., Chen K. (2022). Vision-Related Quality of Life and Association Between Retinal Parameters in Patients with Non-Arteritic Anterior Ischemic Optic Neuropathy. Int. J. Gen. Med..

[37] Keren S., Keren S., Zanolli M., Dotan G. (2017). Visual outcome following bilateral non-arteritic anterior ischemic optic neuropathy: A systematic review and meta-analysis. BMC Ophthalmol..

[38] Kemchoknatee P., Singhakul C., Tangon D., Srisombut T. (2022). Factors Associated with Visual Acuity in Non-arteritic Ischemic Optic Neuropathy Patients: A Five-Year Cross-Sectional Study. Cureus.

[39] Salvetat M.L., Pellegrini F., Spadea L., Salati C., Zeppieri M. (2023). Non-Arteritic Anterior Ischemic Optic Neuropathy (NA-AION): A Comprehensive Overview. Vision.

[40] Sabadia S., Nolan R., Galetta K.M., Narayana K., Wilson J.A., Calabresi P.A., Frohman E.M., Galetta S.L., Balcer L.J. (2016). 20/40 or better visual acuity after optic neuritis: Not as good as we once thought?. J. Neuro-Ophthalmol..

[41] Balcer L.J., Baier M., Kunkle A.M., Rudick R.A., Weinstock-Guttman B., Simonian N.A., Galetta S., Cutter G., Maguire M.G. (2000). Self-reported visual dysfunction in multiple sclerosis: Results from the 25-Item National Eye Institute Visual Function Questionnaire (VFQ-25). Mult. Scler. J..

[42] Galetta K.M., Graves J., Talman L.S., Lile D.J., Frohman E.M., Calabresi P.A., Galetta S.L., Balcer L.J. (2012). Visual pathway axonal loss in benign multiple sclerosis: A longitudinal study. J. Neuro-Ophthalmol..

[43] Jarocki A., Benard-Seguin E., Gonzalez L.A., Costello F., Andrews C.A., Kerber K., De Lott L.B. (2023). Predictors of Long-Term Visual Acuity in a Modern Cohort of Patients with Acute Idiopathic and Multiple Sclerosis-Associated Optic Neuritis. J. Neuro-Ophthalmol..

[44] Dolcetti E., Buttari F., Bruno A., Azzolini F., Gilio L., Di Caprio V., Lauritano G., Borrelli A., Galifi G., Furlan R. (2024). Low-contrast visual acuity test is associated with central inflammation and predicts disability development in newly diagnosed multiple sclerosis patients. Front. Neurol..

