# Supplementary material for: Vision-Related Quality of Life in Patients with Optic Neuropathy: Insights from a Portuguese Single Center Using the NEI-VFQ-25
Source: Neurol Int. 2025 Nov 11;17(11):184. doi: 10.3390/neurolint17110184 (PMC12655186; doi:10.3390/neurolint17110184)
Supplement: Supplementary file 1 [file neurolint-17-00184-s001.zip › Final Supplementary_Table_s1_ON_Subgroups.pdf]

Supplementary Material

Table 1. Number of cases (n) for each etiological subgroup within the Ischemic and Other ON categories.

Category	Subgroup	n
**Ischemic ON**	AAION*	11
**Ischemic ON**	NAION	23
**Other ON**	Toxic	2
**Other ON**	Nutritional	2
**Other ON**	Infectious	3

Note: Statistical comparison between these subgroups was not performed due to small sample sizes.
