# Supplementary material for: Vision-Related Quality of Life in Patients with Optic Neuropathy: Insights from a Portuguese Single Center Using the NEI-VFQ-25
Source: Neurol Int. 2025 Nov 11;17(11):184. doi: 10.3390/neurolint17110184 (PMC12655186; doi:10.3390/neurolint17110184)
Supplement: Supplementary file 1 [file neurolint-17-00184-s001.zip › manual_cm2000.pdf]

Version 2000

The National Eye Institute 25-Item Visual Function Questionnaire (VFQ-25)

Version 2000

This final version of the VFQ-25 differs from the previous version in that it includes an extra driving item from the appendix of supplementary questions as part of the base set of items. Also, the revised scoring algorithm excludes the single-item general health rating question from the calculation of the vision-targeted composite score. Because of these 2 changes, the base set of items actually includes 26 questions, however, only 25 are vision-targeted and included in the composite score. Please see the "Frequently Asked Questions" or FAQ section for additional clarifications of these changes.

Background

The National Eye Institute (NEI) sponsored the development of the VFQ-25 with the goal of creating a survey that would measure the dimensions of self-reported vision-targeted health status that are most important for persons who have chronic eye diseases. Because of this goal, the survey measures the influence of visual disability and visual symptoms on generic health domains such as emotional well-being and social functioning, in addition to task-oriented domains related to daily visual functioning. Questions included in the VFQ-25 represent the content identified during a series of condition-specific focus groups with patients who had age-related cataracts, glaucoma, age-related macular degeneration, diabetic retinopathy, or CMV retinitis. [1]

The VFQ-25 is the product of an item-reduction analysis of the longer field test version of the survey called the 51-item National Eye Institute Vision Function Questionnaire (NEI-VFQ).[2] The longer version contains 51 questions which represent 13 different sub-scales. The NEI-VFQ Field Test Study collected the data needed to examine the reliability and validity of the survey across all of the above-mentioned ocular diseases. Also, reliability and validity was assessed in a heterogeneous group of patients with low vision from any cause and a group of age-matched persons with normal vision. A published report describes the psychometric properties of the longer field test version of the survey. [3] Additional a number of clinical studies have used either the 51 or the 25-item version of the NEI-VFQ across a number of chronic ocular conditions. [4-8] Despite the success of the longer field test version and its continued use, to enhance feasibility a short-form version was planned since the earliest developmental phase.

The VFQ-25 consists of a base set of 25 vision-targeted questions representing 11 vision-related constructs, plus an additional single-item general health rating question. The VFQ-25 also includes an appendix of additional items from the 51-item version that researchers can use to expand the scales up to 39 total items. All items in the VFQ-25 are from the 51-item field test version; no new items were developed for use in the VFQ-25. Unless otherwise specified, the remainder of this document will use the term VFQ-25 to refer to the base set of items.

The VFQ-25 takes approximately 10 minutes on average to administer in the interviewer format. There is also a self-administered version of the survey, however, psychometric testing of the self-administered version has not been done. The VFQ-25 generates the following vision-targeted sub-scales: global vision rating (1), difficulty with near vision activities (3), difficulty with distance vision activities (3), limitations in social functioning due to vision (2), role limitations due to vision (2), dependency on others due to vision (3), mental health symptoms due to vision (4), driving difficulties (3), limitations with peripheral (1) and color vision (1), and ocular pain (2). Additionally,

the VFQ-25 contains the single general health rating question which has been shown to be a robust predictor of future health and mortality in population-based studies. Please see the FAQ section for more information about the general health rating question.

Development of the NEI VFQ-25

The guiding principles for the selection of the short-form items included: 1) low item-level missing data rates; 2) normal distribution of response choices; and 3) retention of items that explained the greatest proportion of variance in the 51-item sub-scales. The items retained in the VFQ-25 and the optional items (provided in the appendix to the survey) are listed on Table 1. A report describing the performance of the VFQ-25 relative to the Field Test version is currently under review. [2] The reliability and validity of the VFQ-25 is similar to that observed for the 51-item version of the survey. On average, each VFQ-25 sub-scale predicts 92% of the variance in the corresponding 51-item sub-scale score.

Optional Items

Appendix 1 consists of additional questions that users may add to a specific sub-scale. Inclusion of these may be helpful if a particular sub-scale represents the primary domain of vision-targeted HRQOL that is felt to be most important for the condition under study. For example, if a user is testing a new treatment for macular degeneration, by adding near vision questions A3, A4, and A5 to VFQ-25 questions 5, 6, and 7, the investigator would have a six-item near vision scale rather than a three-item scale. The addition of these items would enhance the reliability of the near vision sub-scale and is likely to improve the responsiveness of the sub-scale to the intervention over time (Table 6). If items from the appendix are used, the VFQ-25 developers would encourage users to incorporate all optional items for a given sub-scale. This strategy will enhance the comparability of results across studies.

Scoring

Scoring VFQ-25 with or without optional items is a two-step process:

- First, original numeric values from the survey are re-coded following the scoring rules outlined in Table 2. All items are scored so that a high score represents better functioning. Each item is then converted to a 0 to 100 scale so that the lowest and highest possible scores are set at 0 and 100 points, respectively. In this format scores represent the achieved percentage of the total possible score, e.g. a score of 50 represents 50% of the highest possible score.

- In step 2, items within each sub-scale are averaged together to create the 12 sub-scale scores. Table 3 indicates which items contribute to each specific sub-scale. Items that are left blank (missing data) are not taken into account when calculating the scale scores. Sub-scales with at least one item answered can be used to generate a sub-scale score. Hence, scores represent the average for all items in the sub-scale that the respondent answered.

Composite Score Calculation

To calculate an overall composite score for the VFQ-25, simply average the vision-targeted sub-scale scores, excluding the general health rating question. By averaging the sub-scale scores rather than the individual items we have given equal weight to each sub-scale, whereas averaging the items would give more weight to scales with more items.

Table 1. Item Number Translation from the 51-Item Field Test Version to the VFQ 25

S = retained in the VFQ-25, A = retained in the appendix should be used for the VFQ-39,

--- = deleted from the VFQ-25 & VFQ-39

Field Test Version Ques.#	Sub-scale	Status	VFQ-25 Ques. #	Field Test Version Ques.#	Sub-scale	Status	VFQ-25 Ques. #
**1**	general health	S	**1**	**29**	social fx	---	---
**2**	general health	A	**A1**	**30**	social fx	A	**A9**
**3**	general vision	S	**2**	**31**	social fx	S	**13**
**4**	expectations	---	---	**32**	distance vision	A	**A8**
**5**	well-being/ distress	S	**3**	**33**	distance vision	A	**A7**
**6**	well-being/ distress	---	---	**34**	distance vision	S	**14**
**7**	ocular pain	S	**19**	**35**	driving (filter item)	S	**15**
**8**	expectations	---	---	**35a**	driving (filter item)	S	**15a**
**9**	expectations	---	---	**35b**	driving (filter item)	S	**15b**
**10**	expectations	---	---	**35c**	driving	S	**15c**
**11**	well-being/ distress	S	**25**	**36**	driving	---	---
**12**	ocular pain	S	**4**	**37**	driving	S	**16**
**13**	well-being/ distress	---	---	**38**	driving	S	**16a** *
**14**	general vision	A	**A2**	**39a**	role limitations	S	**17**
**15**	near vision	S	**5**	**39b**	role limitations	A	**A11a**
**16**	near vision	A	**A3**	**39c**	well-being/ distress	---	---
**17**	near vision	S	**6**	**39d**	role limitations	---	---
**18**	near vision	---	---	**39e**	role limitations	A	**A11b**
**19**	near vision	S	**7**	**39f**	role limitations	S	**18**
**20**	distance vision	S	**8**	**40**	well-being/ distress	A	**A12**
**21**	distance vision	---	---	**41**	dependency	S	**20**
**22**	distance vision	S	**9**	**42**	well-being/ distress	S	**21**
**23**	peripheral vision	S	**10**	**43**	well-being/ distress	S	**22**
**24**	distance vision	A	**A6**	**44**	dependency	---	---
**25**	social fx	S	**11**	**45**	dependency	A	**A13**
**26**	near vision	A	**A4**	**46**	dependency	S	**23**
**27**	color vision	S	**12**	**47**	dependency	S	**24**
**28**	near vision	A	**A5**				

* VFQ-25 item 16a was listed in previous versions as part of the appendix of supplemental items (#A10).

Table 2. Scoring Key: Recoding of Items

Item Numbers	Change original response category [(a)]	To recoded value of:
1,3,4,15c[(b)]	1	100
	2	75
	3	50
	4	25
	5	0
2	1	100
	2	80
	3	60
	4	40
	5	20
	6	0
5,6,7,8,9,10,11,12,13,14,16,16a	1	100
A3,A4,A5,A6,A7,A8,A9[(c)]	2	75
	3	50
	4	25
	5	0
	6	*
17,18,19,20,21,22,23,24,25,	1	0
A11a,A11b,A12,A13	2	25
	3	50
	4	75
	5	100
A1,A2	0	0
	to	to
	10	100

[(a)] Precoded response choices as printed in the questionnaire.

[(b)] Item 15c has four-response levels, but is expanded to a five-levels using item 15b.
Note: If 15b=1, then 15c should be recoded to “0”
If 15b=2, then 15c should be recoded to missing.
If 15b=3, then 15c should be recoded to missing.

[(c)] “A” before the item number indicates that this item is an optional item from the Appendix. If optional items are used, the NEI-VFQ developers encourage users to use all items for a given sub-scale. This will greatly enhance the comparability of sub-scale scores across studies.

* Response choice "6" indicates that the person does not perform the activity because of non-vision related problems. If this choice is selected, the item is coded as "missing."

Table 3. Step 2: Averaging of Items to Generate VFQ-25 Sub-Scales

Scale	Number of items	Items to be averaged (after recoding per Table 2)
General Health	1	1
General Vision	1	2
Ocular Pain	2	4, 19
Near Activities	3	5, 6, 7
Distance Activities	3	8, 9, 14
Vision Specific:		
Social Functioning	2	11, 13
Mental Health	4	3, 21, 22, 25
Role Difficulties	2	17, 18
Dependency	3	20, 23, 24
Driving	3	15c, 16, 16a
Color Vision	1	12
Peripheral Vision	1	10

Table 4. Step 2: Averaging of Items to Generate VFQ-39 Sub-Scales (VFQ-25 + Optional Items)

Scale	Number of items	Items to be averaged (after recoding per Table 2)
General Health	2	1, A1
General Vision	2	2, A2
Ocular Pain	2	4, 19
Near Activities	6	5, 6, 7, A3, A4, A5
Distance Activities	6	8, 9, 14, A6, A7, A8
Vision Specific:		
Social Functioning	3	11, 13, A9
Mental Health	5	3, 21, 22, 25, A12
Role Difficulties	4	17, 18, A11a, A11b
Dependency	4	20, 23, 24, A13
Driving	3	15c, 16, 16a
Color Vision	1	12
Peripheral Vision	1	10

Figure 1. Example of VFQ-25 Scoring Algorithm for Near Activities Sub-Scale

5. How much difficulty do you have reading ordinary print in newspapers? Would you say you have:

No difficulty at all.. 1
A little difficulty 2
Moderate difficulty 3
Extreme difficulty..(4)
Stopped doing this because of your eyesight 5
Stopped doing this for other reasons or not
interested in doing this.. 6

6. How much difficulty do you have doing work or hobbies that require you to see well up close, such as cooking, sewing, fixing . . . ? Would you say you have:

No difficulty at all..(1)
A little difficulty 2
Moderate difficulty 3
Extreme difficulty.. 4
Stopped doing this because of your eyesight 5
Stopped doing this for other reasons or not
interested in doing this.. 6

7. Because of your eyesight, how much difficulty do you have finding something on a crowded shelf? Would you say you have:

No difficulty at all.. 1
A little difficulty 2
Moderate difficulty 3
Extreme difficulty..(4)
Stopped doing this because of your eyesight 5
Stopped doing this for other reasons or not
interested in doing this.. 6

Scoring example - Figure 1

Items 5, 6, and 7 are used to generate the near activities sub-scale score (Table 3). Each of the items has 6 response choices. Response choice 6 indicates that the respondent does not perform the activity because of reasons that are unrelated to vision. If a respondent selects this choice, the answer is treated as missing and an average of the remaining items is calculated. Response choice 5 indicates that an activity is so difficult that the participant no longer performs the activity. This extremely poor near vision response choice is recoded to "0" points before taking an average of all three items. To score all items in the same direction, Table 2 shows that responses 1 through 5 for items 5, 6, and 7 should be recoded to values of 100, 75, 50, 25, and 0 respectively. If the respondent is missing one of the items, the person's score will be equal to the average of the two non-missing items.

Formula:

$$\text{Mean} = \frac{\text{(Score for each item with a non-missing answer)}}{\text{Total number of items with non-missing answers}}$$

Example:

$$\text{With responses converted:} = \frac{(25 + 100 + 25)}{3} = 50$$

Note: 100 = Best, 0 = Worst possible score.

Psychometric properties of VFQ-25 sub-scales

Psychometric data for VFQ-25 reported in the earlier pre-publication version of the scoring manual have been updated and submitted for peer-reviewed publication.[2] The values reported in this document are identical to those reported in the future publication and should be used when citing the performance characteristics of the VFQ-25.

Statistical Power Calculations

Tables 8, 9, and 10 are provided to estimate statistical power when using the VFQ-25 and VFQ-39. These tables estimate the number of subjects needed per group to attain 80% power (alpha = 0.05, two-tailed) depending on the anticipated difference in scores between groups. Table 8 contains power calculations for changes over time between two experimental (i.e. randomized) groups using a repeated-measures design. For example, if one were interested in being able to detect a 5-point difference for the VFQ-25 General Vision sub-scale, one would need 271 subjects per group. Table 9 shows power calculations for two experimental groups using a single, post-intervention measurement design. Such a design is not as precise as a design that uses a baseline and post-intervention measurement points (i.e., more subjects are needed per group to detect the same difference). Table 10 provides corresponding sample size information for a non-experimental (i.e. non-randomized) repeated-measures design where subjects self-select into the two groups. One sees that the number of subjects needed per group is more than that needed for a randomized experiment (Table 8) and less than the number needed for a randomized, post-intervention-only measurement design (Table 9).

Table 8. Sample sizes needed per group to detect differences in *change over time* between two experimental groups for the VFQ-25, repeated measures design

		Number of Points Difference			
Scale Name	SD	2	5	10	20
**VFQ-25:**					
General Health	26.00	1696	271	68	17
General Vision	21.00	1106	177	44	11
Ocular Pain	17.00	725	116	29	7
Near Activities	29.00	2110	338	84	21
Distance Activities	29.00	2110	338	84	21
Social Functioning	27.00	1829	293	73	18
Mental Health	27.00	1829	293	73	18
Role Difficulties	29.00	2110	338	84	21
Dependency	28.00	1967	315	79	20
Driving	35.00	3073	492	123	31
Color Vision	23.00	1327	212	53	13
Peripheral Vision	27.00	1829	293	73	18
VFQ-25 Composite	20.00	1004	161	40	10
**VFQ-39:**					
General Health	21.00	1106	177	44	11
General Vision	19.00	906	145	36	9
Ocular Pain	17.00	725	116	29	7
Near Activities	28.00	1967	315	79	20
Distance Activities	26.00	1696	271	68	17
Social Functioning	25.00	1568	251	63	16
Mental Health	26.00	1696	271	68	17
Role Difficulties	28.00	1967	315	79	20
Dependency	27.00	1829	293	73	18
Driving	35.00	3073	492	123	31
Color Vision	23.00	1327	212	53	13
Peripheral Vision	27.00	1829	293	73	18
VFQ-39 Composite	21.00	1106	177	44	11

Note: Scales are all scored on 0-100 possible range. Estimates assume alpha = 0.05, two-tailed t-test, power = 80%, and an inter-temporal correlation between scores of 0.60.

Table 9. Sample sizes needed per group to detect differences between two experimental groups for the VFQ-25, *post-intervention measures only*.

		Number of Points Difference			
Scale Name	SD	2	5	10	20
**VFQ-25:**					
General Health	26.00	2650	424	106	26
General Vision	21.00	1729	277	69	17
Ocular Pain	17.00	1133	181	45	11
Near Activities	29.00	3297	527	132	33
Distance Activities	29.00	3297	527	132	33
Social Functioning	27.00	2858	457	114	29
Mental Health	27.00	2858	457	114	29
Role Difficulties	29.00	3297	527	132	33
Dependency	28.00	3073	492	123	31
Driving	35.00	4802	768	192	48
Color Vision	23.00	2074	332	83	21
Peripheral Vision	27.00	2858	457	114	29
VFQ-25 Composite	20.00	1568	251	63	16
**VFQ-39:**					
General Health	21.00	1729	277	69	17
General Vision	19.00	1415	226	57	14
Ocular Pain	17.00	1133	181	45	11
Near Activities	28.00	3073	492	123	31
Distance Activities	26.00	2650	424	106	26
Social Functioning	25.00	2450	392	98	25
Mental Health	26.00	2650	424	106	26
Role Difficulties	28.00	3073	492	123	31
Dependency	27.00	2858	457	114	29
Driving	35.00	4802	768	192	48
Color Vision	23.00	2074	332	83	21
Peripheral Vision	27.00	2858	457	114	29
VFQ-39 Composite	21.00	1729	277	69	17

Note: Scales are all scored on 0-100 possible range. Estimates assume alpha = 0.05, two-tailed t-test, and power = 80%.

Table 10. Sample sizes needed per group to detect differences between two *self-selected groups* for the VFQ-25, repeated measures design

		Number of Points Difference			
Scale Name	SD	2	5	10	20
**VFQ-25:**					
General Health	26.00	2120	339	85	21
General Vision	21.00	1383	221	55	14
Ocular Pain	17.00	906	145	36	9
Near Activities	29.00	2637	422	105	26
Distance Activities	29.00	2637	422	105	26
Social Functioning	27.00	2286	366	91	23
Mental Health	27.00	2286	366	91	23
Role Difficulties	29.00	2637	422	105	26
Dependency	28.00	2459	393	98	25
Driving	35.00	3842	615	154	38
Color Vision	23.00	1659	265	66	17
Peripheral Vision	27.00	2286	366	91	23
VFQ-25 Composite	20.00	1254	201	50	13
**VFQ-39:**					
General Health	21.00	1383	221	55	14
General Vision	19.00	1132	181	45	11
Ocular Pain	17.00	906	145	36	9
Near Activities	28.00	2459	393	98	25
Distance Activities	26.00	2120	339	85	21
Social Functioning	25.00	1960	314	78	20
Mental Health	26.00	2120	339	85	21
Role Difficulties	28.00	2459	393	98	25
Dependency	27.00	2286	366	91	23
Driving	35.00	3842	615	154	38
Color Vision	23.00	1659	265	66	17
Peripheral	27.00	2286	366	91	23
VFQ-39 Composite	21.00	1383	221	55	14

Note: Scales are all scored on 0-100 possible range.Estimates assume alpha = 0.05, two-tailed t-test, power = 80%, and an inter-temporal correlation between scores of 0.60.

Frequently Asked Questions (FAQ)

Q. *What kind of permissions are required to use the VFQ-25 in a research study?*

The VFQ-25 is a public document available without charge for all researchers to use provided they identify the measure as such in all publications and cite the appropriate developmental papers. Users do not need to notify the developers or the NEI that they intend to use the measure. However, there are some specific permissions for using the VFQ-25 that are detailed on the cover page of the questionnaire itself. These include acknowledging in all publications that the VFQ-25 was developed by RAND and funded by the NEI, and that any changes made to the measure for your particular study will be identified as such.

Q. *Can I change the format of the VFQ-25 to suit my study?*

Any change to the wording or order of the items would constitute a change to the measure and should be specified as such in any published papers. Other than this, it is expected that researchers may need to change the format or appearance of items to suit their purposes.

As of August 2000, to our knowledge no studies have reported on the effect of item order on responses to VFQ-25 or other similar vision-targeted surveys. That is, whether responses change depending where particular items appear in the questionnaire. However, to ensure the comparability of scores across studies, it is our position that the order of items should not be changed.

Q. Has the VFQ-25 been translated into any other languages?

As of August 2000, the developers are aware of translation into approximately 9 languages. For the cost of distribution, a Spanish language version for Mexican-American populations is available from the UCLA and RAND based developers. The developers will provide researchers with the names of other persons to contact for other language translations. Should researchers wish to translate the VFQ-25, the same permissions apply, with the additional requirement that all publications specify responsibility for the translation along with instructions for obtaining a copy of the translated version.

Q. Do you have any additional normative information for specific populations?

The developers currently are not conducting studies for the express purpose of further investigating the psychometric properties of the VFQ-25 or producing normative data. However, many researchers are currently using the VFQ-25 as an endpoint or outcome in a number of health services and clinical studies. It is likely that as these studies are completed, results that are relevant to better understanding the performance of the VFQ-25 will accompany the main results of each study. The developers and staff at the NEI are aware of other researchers who are collecting condition-specific normative data on population-based samples with the VFQ-25 and when possible will provide contact information for these investigators to new users.

Q. How relevant is the normative data provided in the scoring manual to my sample?

The means, standard deviations, and statistical power values shown in this document were estimated using cross-sectional data from the Field Test Study. Participants recruited for the Field Test were not randomly sampled, but rather were identified for enrollment based on clinical criteria biased towards persons with moderate to severe forms of each target disease. Further, because it was our desire to enroll a broad spectrum of patients based on disease severity, we did not take into consideration treatment status. Please see references #3 for a full

description of the NEI-VFQ field test study sample.

Q. Why is a single-item general health item included in the VFQ-25?

During the developmental phase of the NEI-VFQ, vision-targeted health-related quality of life (HRQOL) was a relatively new concept. For this reason, we included this question to insure that researchers had a minimal amount of information about a person's general health status to use as a benchmark against other published samples or cohorts.

This general health rating question has been widely used in studies and is a robust predictor of future health and mortality. However, to fully measure generic HRQOL, many quality of life measurement experts recommend including a separate generic measure of HRQOL such as the SF-36 or SF-12.[9] In such a situation the single-item VFQ-25 general health rating question is not needed because the identical question is asked as part of these surveys.[10, 11]

Q. Should we be looking at the sub-scales or the composite score?

The VFQ-25 sub-scales are grouped by theme or domain. So, for example, items having to do with near vision are differentiated from items having to do with other vision activities like distance vision or ocular pain. This does not mean that the items are not highly correlated or that they are psychometrically distinct. What it does mean is that researchers should beforehand carefully consider which vision-specific domains are most likely to be influenced by a particular disease and/or treatment and then focus on the results from those sub-scales to support their findings.

The composite score is best used in situations where an overall measure of vision-targeted health related quality of life is desired. For example, in studies where it is not clear what the specific impact of ocular disease or a new treatment might be. Also, in situations where differences can be hypothesized between groups beforehand across multiple sub-scales but the overall sample size of the study is relatively small, because it is likely that the error term for the composite score is likely to be smaller than for any given sub-scale, it may be more efficient to represent these differences as a single score.

Q. What benefit is there to using the VFQ-25 over a measure more specific to a particular disease, like the Activity of Daily Vision Scale (ADVS)[10] for persons with age-related cataracts?

The VFQ-25 contains items that are very similar to items found in other vision-targeted measure like the ADVS that are more task oriented. However, whereas the ADVS was designed specifically to assess a set of activities most relevant to patients undergoing cataract surgery, the VFQ-25 expands the range of activities to measure the impact of ocular disease on broader domains of health such as social and emotional well-being. Serious ocular diseases that lead to irreversible loss of vision are likely to impact dimensions of a person's life beyond simple tasks such as driving or reading the newspaper, and similarly, by preserving vision, many successful interventions also will impact persons' lives at this more global level. Especially in these situations, use of the VFQ-25 should be considered.

Q. Why does the response to item 15b, "stopped driving due to vision <u>and</u> other reasons", generate a missing score for the subsequent driving items?

Driving items 15, 15a, and 15b are filter questions designed to specify whether a person has ever driven a car, and if so, whether they are currently driving or if they have stopped. If people have never driven a car, then, of course, their answers should be set to missing for all driving items. Similarly, this also applies to people who have stopped driving for other reasons not due to vision. However, in the course of pilot testing the field test participants wanted this additional mixed response option. It was our decision that although persons did indeed report

not driving due to vision, it was not clear how much of a role the "other" reason also played in this decision. Therefore, we set the scoring criteria for this response to be missing for all subsequent driving items to be absolutely sure that all driving responses reflected only problems with vision. Should researchers wish to change this response option to allow persons to answer subsequent driving items (currently there is a skip to item #17), this change should be noted in subsequent publications.

References

1. Mangione CM, Berry S, Lee PP, et al. Identifying the content area for the National Eye Institute Vision Function Questionnaire (NEI-VFQ): Results from focus groups with visually impaired persons. *Arch Ophthalmol.* 1998;116:227-238.

2. Mangione CM, Lee PP, Gutierrez PR, et al. Development of the 25-item National Eye Institute Visual Function Questionnaire (VFQ-25). *Arch Ophthalmol.* 2001 (in press).

3. Mangione CM, Lee PP, Pitts J, et al. Psychometric properties of the National Eye Institute Visual Function Questionnaire, the NEI-VFQ. *Arch Ophthalmol*. 1998;116:1496-1504

4. Gutierrez P, Wilson MR, Johnson C, Gordon M, Cioffi GA, Ritch R; Sherwood M, Meng K, Mangione CM. Influence of glaucomatous visual field loss on health-related quality of life. *Arch Ophthalmol*. 1997;115:777-84.

5. Parrish RK 2nd, Gedde SJ, Scott IU, Feuer WJ, Schiffman JC, Mangione CM, Montenegro-Piniella A. Visual function and quality of life among patients with glaucoma. *Arch Ophthalmol*. 1997;115:1447-55

6. Quality of life assessment in the collaborative ocular melanoma study: design and methods. COMS-QOLS Report No. 1. COMS Quality of Life Study Group. *Ophthal. Epidemiology*. 1999;6:5-17.

7. Scott IU, Smiddy WE, Schiffman J, Feuer WJ, Pappas CJ. Quality of life of low-vision patients and the impact of low-vision services. *Amer. J. Ophthalmol*. 1999;128:54-62.

8. Cole SR, Beck RW, Moke PS, Gal RL, Long DT. The National Eye Institute Visual Function Questionnaire: experience of the ONTT. Optic Neuritis Treatment Trial. *Invest Ophthalmol Vis Sci* 2000;41:1017-21.

9. Mangione CM, Lee PP, Hays RD. Measurement of visual functioning and health-related quality of life in eye disease and cataract surgery. In B. Spilker (ed.), Quality of Life and Pharmacoeconomics in clinical trials, 2nd edition. New York: Raven Press 1996:1045-1051.

10. Hays RD, Sherbourne CD, Mazel RM. The RAND 36-item Health Survey 1.0. *Health Econ* 1993;2:217-227.

11. Ware J Jr, Kosinski M, Keller SD 12-Item Short-Form Health Survey: construction of scales and preliminary tests of reliability and validity. *Med Care* 1996;34:220-33A.

Attachments include:

NEI VFQ-25 (IA = Interviewer-Administered format)
(SA = Self-Administered format)
